# Influence of Basalt/Polypropylene Fiber on Permeability and Uniaxial Compressive Properties of Waste Tire Rubberized Concrete

**DOI:** 10.3390/ma16020481

**Published:** 2023-01-04

**Authors:** Dan-yang Su, Jian-yong Pang, Chen-yue Han, Jian-yu Huang, Xiu-yue Hu, Wei Shi

**Affiliations:** School of Civil Engineering and Architecture, Anhui University of Science and Technology, Huainan 232001, China

**Keywords:** rubber, concrete, basalt fiber, polypropylene fiber, stress-strain relationship, energy dissipation

## Abstract

The rubber particles obtained from the grinding of waste tires can replace a portion of the fine aggregates in concrete, thus effectively reducing the level of environmental damage and saving resources. However, when concrete is mixed with rubber, it greatly reduces its strength. In this study, by introducing basalt fiber (BF) and polypropylene fiber (PF) as modified materials in rubberized concrete, the influence of the fiber type/volume ratio on the slump, water absorption, static uniaxial compression, and permeability of the rubberized concrete was tested. The axial compression stress–strain relationship was analyzed, the effect of the fiber type/volume ratio on the energy dissipation of the rubberized concrete during uniaxial compression was expounded, and a stress–strain constitutive model under uniaxial compression was established. The test results showed that the fiber reduces the fluidity and water absorption of the rubberized concrete. Compared with the polypropylene fiber, the basalt fiber increased the strength of the rubberized concrete, while the polypropylene fiber mainly inhibited the expansion and penetration of the macroscopic crack of the rubberized concrete. The mixing of the basalt fiber and polypropylene fiber significantly decreased the release rate of the elastic strain energy of the rubberized concrete, increased the dissipation energy, and thus improved its ductility and toughness. During a loading process under confining pressure, the permeability of the tested specimen decayed exponentially, and the fiber greatly enhanced the anti-permeability of the rubber concrete.

## 1. Introduction

With the rapid development of the global economy and society, the demand for concrete, the most commonly used building material worldwide, has risen sharply. Among them, natural river sand is one of the important raw materials for the preparation of concrete. However, long-term over-exploitation has led to the depletion of natural river sand resources, riverbed collapse, and a reduction in groundwater levels, which has ultimately seriously damaged the ecological environment [1,2]. Therefore, it is necessary to seek a cheap, convenient, and sustainable material that can replace natural river sand. In recent years, cars have contributed a great degree of convenience to the public’s travel, inducing a yearly increase in the output of waste tires. As waste tires are not easy to degrade, regardless of whether they are buried, burned, or stacked< they will seriously pollute the environment and are also unsustainable [3,4,5,6]. Waste tire recycling, which consists of reasonable application in concrete instead of natural river sand, can not only effectively solve the accumulation of waste tires and reduce environmental pollution, but can also reduce the exploitation of natural river sand resources, thus promoting sustainable development.

At present, many scholars continue to study the effect of rubber as a fine aggregate on concrete. For instance, researchers have compared and analyzed the compressive strength and splitting tensile strength of rubber-aggregate concrete and ordinary concrete, finding that, compared with ordinary concrete, the compressive strength of rubber-aggregate concrete was reduced by a maximum of 85% and the splitting tensile strength was reduced by a maximum of 50% [7,8,9,10,11]. Liu et al. [12] studied the fatigue properties of rubberized concrete with different contents, and the results showed that its fatigue resistance was better than that of ordinary concrete, with the greatest fatigue performance found when the rubber content was 20%. Some researchers [13,14,15,16] have found that rubberized concrete has advantages over ordinary concrete, including its lighter weight and lower thermal conductivity. Xu et al. [17] found that, in the state of biaxial compression, although rubber does not transform the macroscopic failure pattern of concrete, rubber concrete not only develops slower cracks during failure compared to ordinary concrete, but also most of the crack width reduction compared to normal concrete is small, showing better plastic damage failure characteristics. Feng et al. [18] conducted dynamic mechanical performance tests on rubberized concrete with different contents, and the results showed that its energy dissipation capacity was better than that of normal concrete at a high strain rate, suggesting that the replacement rate of rubber should not exceed 30%. Gupta et al. [19] studied the workability of rubberized concrete and found that, with an increase in the rubber content, the workability of the concrete gradually decreased. Richardson et al. [20,21,22,23] systematically studied the influence of rubber on concrete under low-temperature environment. The results show that rubber is beneficial in improving the frost resistance and delaying the deterioration of concrete.

Although rubber concrete has economic and environmental advantages compared to ordinary concrete, its greatest advantages are its fatigue resistance, weight, thermal conductivity, failure characteristics, and freeze–thaw resistance. However, the compressive strength of concrete is significantly reduced after the addition of rubber; thus, the safety of the concrete structure is not effectively guaranteed. Therefore, it is necessary to study and improve the strength of rubber concrete. In recent years, the introduction of fibers in concrete to enhance its strength has received extensive attention. In particular, the addition of fibers of different sizes or types can reduce the width of concrete cracks at different structural scales and significantly reinforcement the concrete strength [20,21,22,23,24,25,26,27,28].

Basalt fiber (BF) is a continuous fiber made of basalt rock melted at 1450~1500 °C and drawn at a high speed through platinum-rhodium alloy wire drawing bushing. It belongs to green fiber. The elastic modulus of basalt fiber can reach 93~115 GPa; its tensile strength can reach 200~5000 MPa; and it has a good bonding performance with cement-based materials [29,30]. Polypropylene fiber (PF) has a relatively low elastic modulus and tensile strength and is a flexible fiber. However, it has a high resistance to deformation. When polypropylene fibers are incorporated in concrete, they can decidedly reduce the expansion of concrete macroscopic cracks as well as increase the concrete ductility [31,32].

In view of the fact that rubber will significantly reduce the strength of concrete, in order to ensure the safety of rubber concrete buildings, it is designed to mix basalt fiber and polypropylene fiber in rubber concrete to prepare a new type of green and sustainable concrete (basalt–polypropylene fiber rubber concrete; BPRC). The slump and water absorption of BPRC were measured, and uniaxial compression and permeability tests under different confining pressures were carried out. The strengthening mechanism of basalt fiber (BF) and polypropylene fiber (PF) on rubber concrete was studied systematically.

## 2. Materials and Methods

### 2.1. Raw Materials

The cementitious material used in the present study was a common Portland cement with a compressive strength of 42.5 MPa at 28 days. The coarse aggregate was made of natural crushed stone with a particle size of 5~20 mm, while the fine aggregate was natural river sand with a fineness modulus of 2.65 and apparent density of 2345 kg/m^3^. The rubber was obtained by crushing waste tires with a particle size of 1~2 mm and an apparent density of 1250 kg/m^3^. The main performance parameters of the basalt fiber and polypropylene fiber are shown in Table 1. The water was tap water. The water-reducing agent was an HPWR liquid-type high performance water-reducing agent, and the water-reducing rate was 37%.

### 2.2. Mixture Ratio Design and Specimen Preparation

In order to maximize the use of the waste tires, this study fixed the rubber content at an equal volume to supersede 20% of the fine aggregate. The total volume ratio of the basalt fiber and polypropylene fiber was fixed at 1.5%, and the volume ratio ratio (*V*_BF_:*V*_PF_) was 1:0, 0:1, 1:2, 1:1, and 2:1. Using the absolute volume method [33], after many times in the laboratory trial, the final mix design was determined, as shown in Table 2.

**Figure 1:**
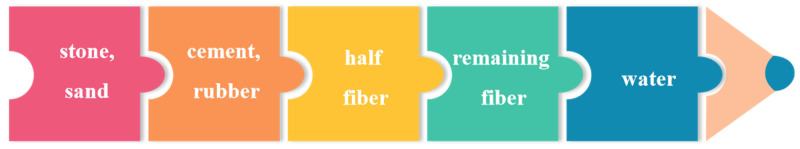
Raw material delivery sequence.

A single-axis horizontal mixer was used to mix the concrete mixture. As shown in Figure 1, the raw material was first mixed with stones and sand for 2 min and then poured into the cement and rubber for another 2 min. The fiber was evenly sprinkled two times, each time sprinkling the fiber for 2 min. Finally, the water-reducing agent was evenly mixed in the water and poured into a blender (Shanghai Shengli Machinery Co., Ltd., Shanghai, China) for 2 min. Then, the concrete mixture was poured into a mold, then vibrated and compacted on a vibrating table. It was removed from the mold after 24 h and placed in a saturated calcium hydroxide solution for a curing period of 28 days.

### 2.3. Test Plan

#### 2.3.1. Slump Test

First, the inner wall of the slump cylinder and the bottom plate were wet. Then, the bottom plate was placed on a level surface, and the slump cylinder was placed in the center of the bottom plate. The mixed concrete was put in the slump cylinder three times, and a tamper was used to pound it evenly 25 times after each time. After removing the concrete at the bottom of the cylinder, the slump cylinder was lifted vertically. When the concrete was not slumping, the distance between the cylinder height and the highest point of the concrete was tested, which comprised the slump value of the group of concrete.

#### 2.3.2. Water Absorption Test

The water absorption was measured on the 7th, 14th, and 28th days of curing using the calculations shown in Equation (1):

(1) $$W_{a}=\frac{m_{s}-m_{d}}{m_{d}}\times 100\%$$

where *W_a_* is the water absorption; *m_s_* is the saturated mass; and *m_d_* is the drying quality.

#### 2.3.3. Uniaxial Compression Test

The specimens were cylindrical with a diameter of 100 mm and a height of 200 mm. The test equipment used in this study was the WAW-2000D electro-hydraulic servo universal testing machine (Jinan United Testing Technology Co., Ltd., Jinan, China). A quasi-static loading process was simulated using the displacement-controlled loading method at a speed of 0.5 mm/min. During the test, the longitudinal deformation and axial force of the test block were collected automatically by the test system, and the displacements at both ends of the test block were measured by the displacement meter. Before the test, the two end surfaces of the test block were ground smooth and lubricated. Before the formal loading, each specimen was preloaded at a loading rate of 0.5 mm/min. When processing the results, three typical test stress–strain curves were selected from each group. Their average stress–strain curves were obtained through data processing, and the average curve was used as the analysis curve of this group of tests.

#### 2.3.4. Permeability Test

The specimens were saturated with water the day before the test. The test equipment used in the present study was the TAW-2000 triaxial testing machine (Changchun Chaoyang Test Instrument Co., Ltd., Changchun, China). The confining pressure was set to C = 3, 4, 5, 6, 7, and 8 MPa; the osmotic water pressure was set at a constant of 1.6 MPa; and the axial pressure was set at a constant of 1 kN. The calculation method used for determining the permeability is shown in Equation (2):

(2) $$K=\frac{\mu QL}{AP}$$

where *K* is the permeability; *μ* is the viscosity coefficient of the water; *Q* is the flow rate of the water infiltrating the specimen; *L* is the height of the specimen; *A* is the cross-sectional area of the water passing through; and *p* is the osmotic water pressure.

## 3. Test Results and Analysis

### 3.1. Slump and Water Absorption

**Figure 2:**
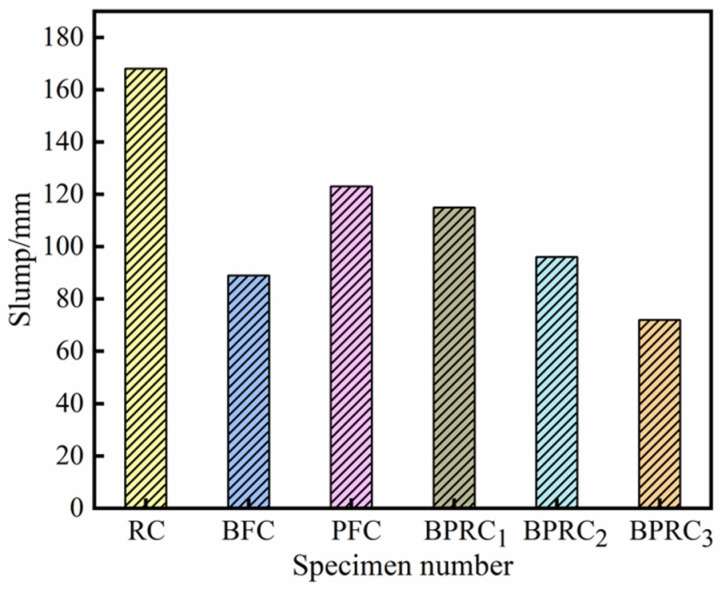
The slump of each group of specimens.

The influence of the fiber type/volume ratio on the slump of the rubber concrete is shown in Figure 2. It can be seen that the slump of the fiber–rubber concrete is lower than that of the RC, indicating that the fiber reduces the fluidity of the rubber concrete. The slump of the BFC is lower than that of the PFC, and, in BPRC_1_, BPRC_2_, and BPRC_3_, the slump reduction with an improve in the basalt fiber volume fraction, indicating that the basalt fiber has a greater influence on the slump of the rubber concrete than the polypropylene fiber.

**Figure 3:**
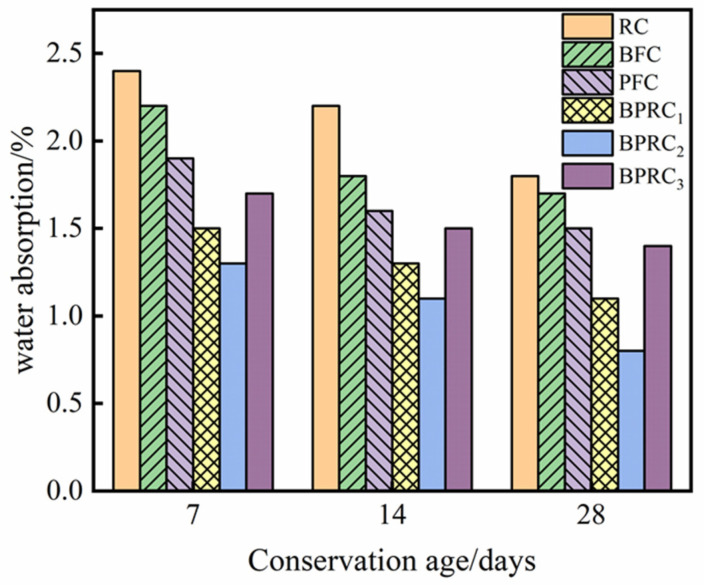
Water absorption of each group of specimens.

The water absorption of each group of specimens at the different curing ages is shown in Figure 3. It can be seen that, with the increase in the curing age, the water absorption rate of the specimens in each group gradually decreases. At the same curing age, the water absorption of the fiber–rubber concrete is lower than that of the RC, which is mainly because the fiber is distributed in the matrix of the rubber concrete, improving its overall compactness. This then leads to the pores and micro-cracks in the matrix becoming effectively blocked, resulting in the decrease in the water absorption. The water absorption rate of the PFC is lower than that of the BFC, and, in BPRC_1_, BPRC_2_, and BPRC_3_, the water absorption rate decreases with the increase in the volume fraction of the polypropylene fiber, indicating that the influence of the polypropylene fiber on the water absorption rate of the rubber concrete is greater than that of the basalt fiber.

### 3.2. Uniaxial Compression Test

#### 3.2.1. Stress–Strain Curve

**Figure 4:**
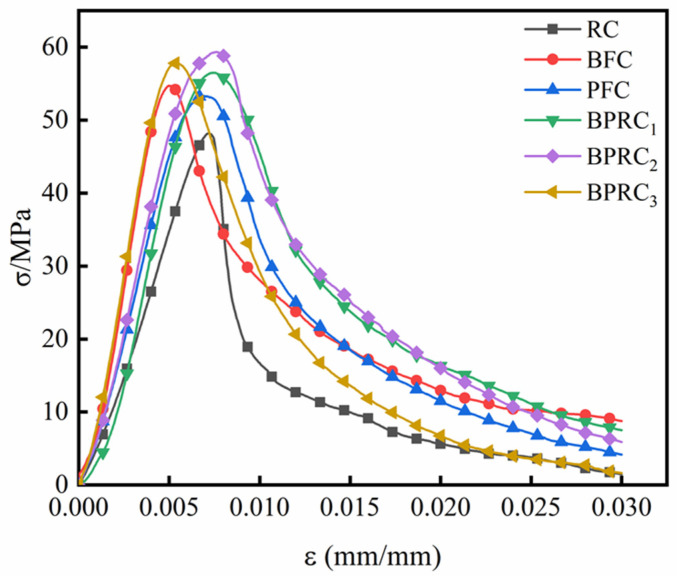
Uniaxial compression stress-strain curve.

The stress–strain curve from the uniaxial compression test is shown in Figure 4, from which the following results could be obtained:

(1) On the whole, the stress–strain curve of the fiber–rubber concrete is obviously fuller than that of the rubber concrete. At the initial stage of loading, the stress–strain curve is close to a straight line, and, at this stage, the slope of the fiber–rubber concrete is obviously larger than that of the rubber concrete. This shows that the fiber can effectively increase the stiffness of the rubber concrete and has a good elasticity. Because of the bridging effect of the fiber, the internal defects of the rubber concrete are reduced, and the elastic stage of fiber–rubber concrete is longer than that of the rubber concrete. Intuitively, the BPRC_3_ group and BFC group revealed similar performances in the elastic stage, the longest elastic time, and the largest slope, which indicated that the basalt fiber in the elastic stage of the rubber concrete lifting effect is more obvious than the polypropylene fiber.
(2) In the stage of rapid stress growth, the stress–strain relationship curve begins to bias from the straight line, with the growth rate of the strain greater than that of the stress at this stage. The slope of the curve decreased gradually, but the decreasing rate was different. The slope of the RC group is the smallest, with those of the BPRC_3_ group and BFC group being the largest. Compared with the RC group, the top of the fiber–rubber concrete curve is more rounded, while the PFC group is the most rounded, indicating that the polypropylene fiber is more conducive to improving the toughness of the rubberized concrete than the basalt fiber. It can be seen from the whole rising stage of the stress–strain curve that the basalt fiber and polypropylene fiber are both beneficial in improving the compressive capacity and deformation capacity of the rubber concrete.
(3) After the peak stress, the stress drops rapidly to the “inflection point” of the curve. Following the inflection point, the stress decreases slowly, and the stress–strain curve becomes convex toward the strain axis until ultimately reaching stability. After the peak stress, the stress of the RC group decreases rapidly, displaying obvious brittle failure, and the decline rate of the fiber–rubber concrete becomes significantly lower than that of the RC group, indicating that the fiber was beneficial in improving the ductility of the rubberized concrete and improving the brittle failure. Among them, the BPRC_2_ group declined the most slowly, mainly because the synergistic effect of the basalt fiber and polypropylene fiber improved the internal defects of the rubberized concrete so that it still had a better bearing capacity after compression failure.

#### 3.2.2. Peak Stress and Elastic Modulus

**Figure 5:**
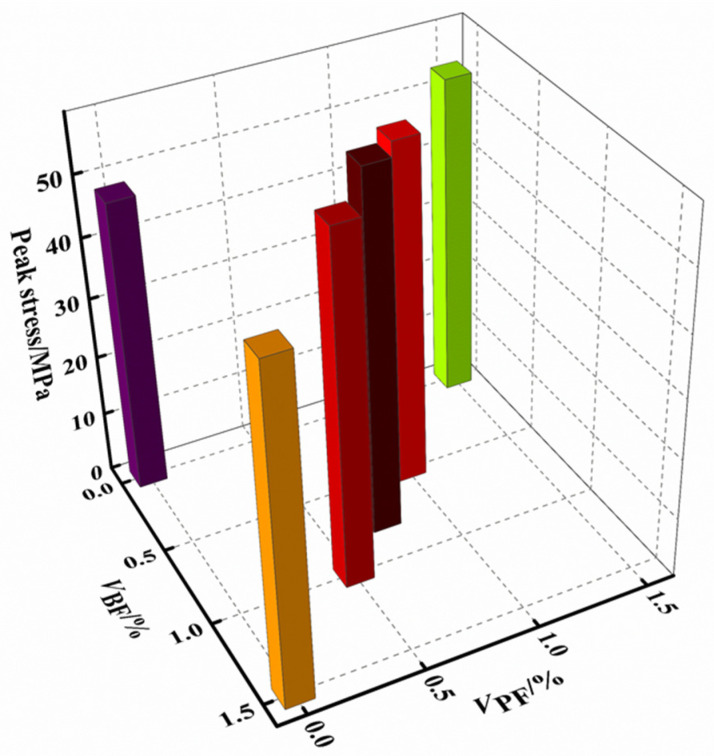
Peak stress.

The effect of the fiber type/volume ratio on the peak stress of the rubberized concrete is shown in Figure 5. It can be seen that the peak stress of the fiber–rubber concrete is larger than that of the rubber concrete. The fiber type/volume ratio has a significant effect on the peak stress of the rubber concrete, with the effect order by fiber type being basalt fiber > polypropylene fiber. When *V*_BF_:*V*_PF_ = 1:1, the peak stress is the largest, i.e., 23.24% higher than that of the rubberized concrete.

**Figure 6:**
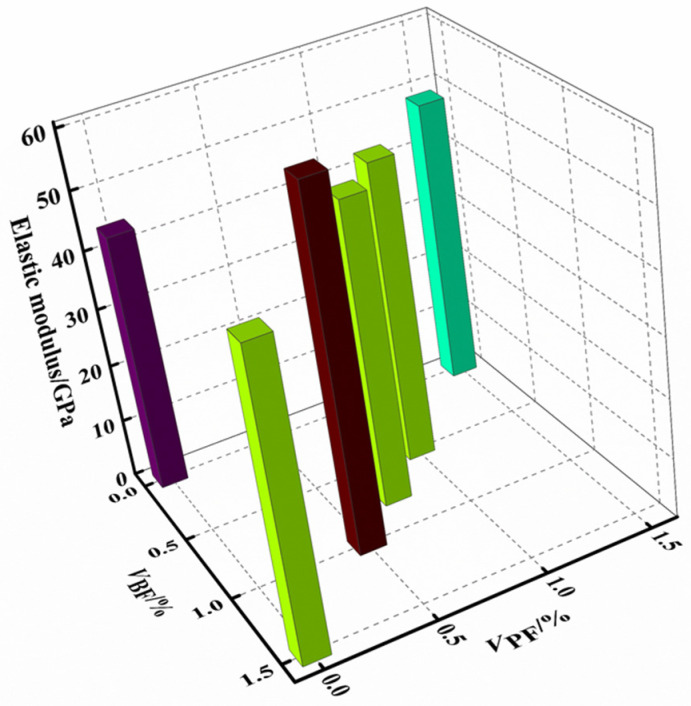
Elastic modulus.

The effect of the fiber type/volume ratio on the elastic modulus of the rubber concrete is shown in Figure 6. As can be seen, the elastic modulus of the fiber–rubber concrete is larger than that of the rubber concrete, and the influence of the polypropylene fiber on the elastic modulus of the rubber concrete is limited, with the elastic modulus of the PFC group increasing by only 10.36% when compared with the RC group. Using a nonlinear regression, the functional relationship between the elastic modulus and the fiber eigenvalues could be obtained as shown in Equation (3):

(3) $$E_{c}=22.91+22.91\times (1+1.19V_{BF})\times (1-0.36V_{PF})$$

where *E_c_* is the elastic modulus.

To sum up, if only from the perspective of improving the peak stress and elastic modulus of rubber concrete, the volume ratio of the basalt fiber should be appropriately increased.

#### 3.2.3. Energy Dissipation Law

The essential characteristic of the physical change process of matter is the transformation of energy [34]. The compression failure of concrete under a static force is essentially a process of energy evolution inside the concrete and energy exchange with the outside world. Under a state of pressure, the input energy is gradually transformed into elastic strain energy, dissipative energy, electromagnetic energy, radiation energy, and so on. Since the electromagnetic energy and radiant energy are negligible under static action, the energy conversion relation of a concrete specimen under compression is represented by Equation (4):

(4) $$U=U_{e}+U_{d}$$

where *U* is the total strain energy per unit volume of the external force input; *U_e_* is the elastic strain energy stored in the concrete interior per unit volume; and *U_d_* is the dissipated energy per unit volume of concrete damage development.

According to the stress–strain relationship, the calculation of the total strain energy, elastic strain energy, and dissipation energy under the unit volume of the external force input can be conducted using Equation (5):

(5) $$\begin{array}{l} U=\int \sigma d\varepsilon \\ U_{e}=\frac{\sigma^{2}}{2E}\\ U_{d}=U-U_{e} \end{array}$$

where *σ* is the stress; *ε* is the strain; and *E* is the initial elastic modulus.

**Figure 7:**
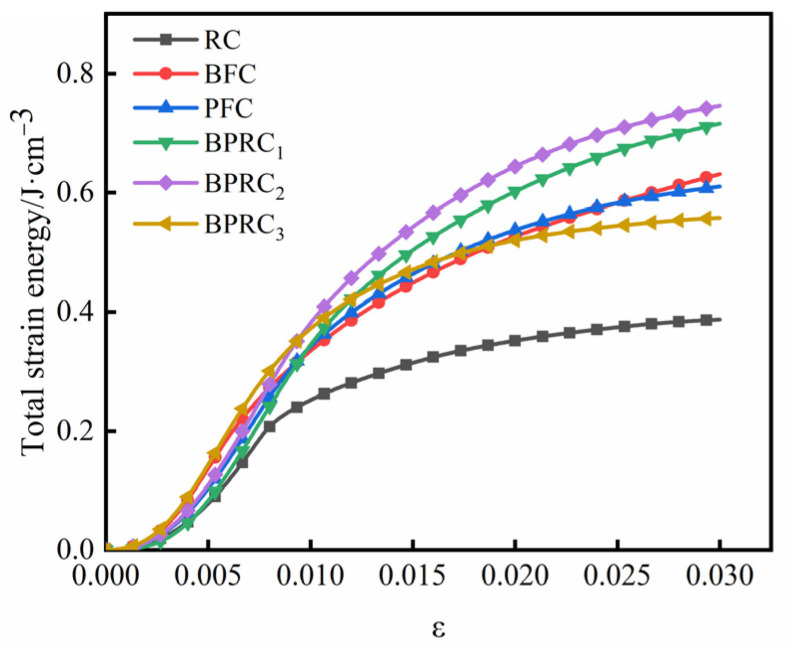
Total strain energy.

**Figure 8:**
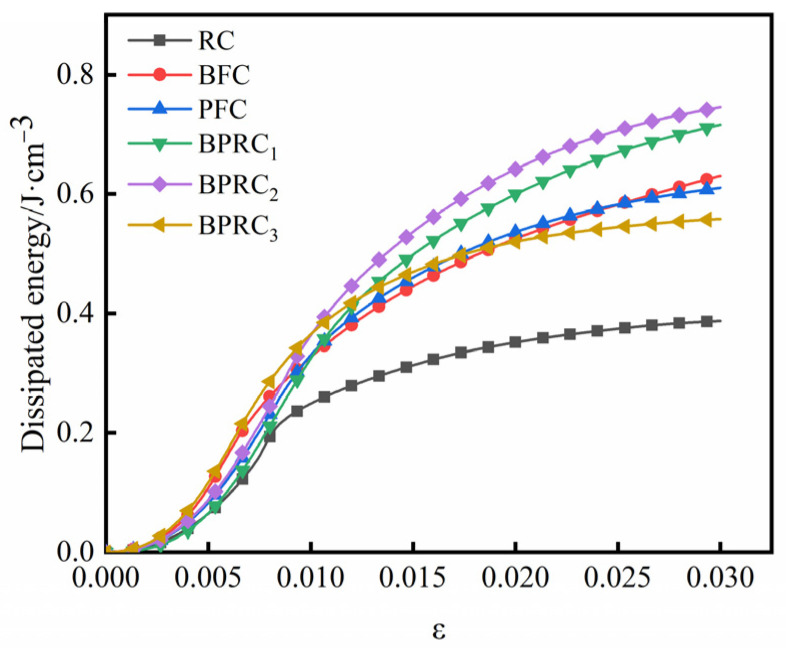
Dissipated energy.

**Figure 9:**
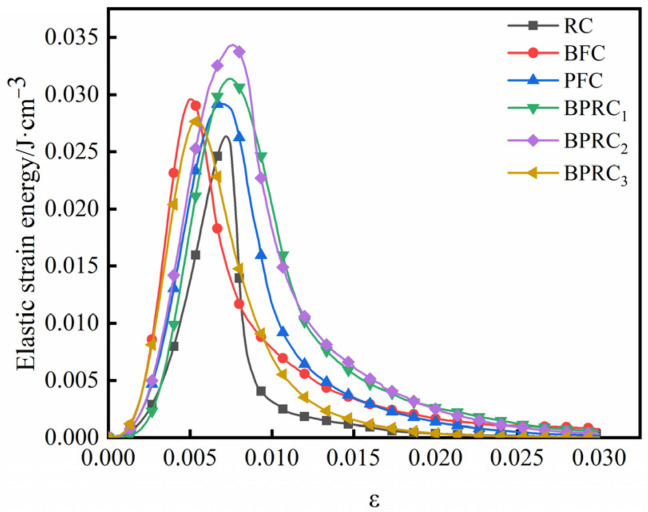
Elastic strain energy.

The total strain energy, dissipative energy, and elastic strain energy of each group of specimens in the process of compression failure are shown in Figure 7, Figure 8 and Figure 9, respectively.

It can be seen from Figure 7 and Figure 8 that the variation laws of the total strain energy and dissipation energy are similar. In the initial deformation stage, the fiber has little effect on the total strain energy and dissipation energy of the rubber concrete. However, with the continuous increase in the load, the total strain energy and dissipation energy of the fiber–rubber concrete are obviously larger than those of the rubber concrete, e.g., the total strain of the BPRC_2_ group maximum energy and dissipated energy. The total strain energy and dissipation energy of the BFC group are slightly larger than those of the PFC group. When the specimen was finally damaged under compression, compared with the RC group, the total strain energy of the BFC group, PFC group, and BPRC2 group increased by 62.98%, 57.62%, and 92.55%, respectively, and the dissipated energy increased by 62.79%, 57.58%, and 92.47%, respectively.

It can be seen from Figure 9 that fibers are beneficial in improving the elastic strain energy of rubber concrete, mainly in terms of two aspects: First, before the peak elastic strain energy, compared with the RC group, the fiber increases the increasing rate of the elastic strain energy of the rubber concrete. Second, after the peak elastic strain energy, the elastic strain energy of the RC group decreases rapidly, while the reduction rate of the elastic strain energy of the fiber–rubber concrete gradually decreases, leading to obvious ductile failure characteristics. Compared with the RC group, the BFC and BPRC_3_ groups had the greatest increasing rate in the elastic strain energy before the peak elastic strain energy, indicating that basalt fiber is more advantageous than polypropylene fiber in increasing the rate of elastic strain energy of rubber concrete. Compared with the RC group, the BPRC_2_ group displayed the smallest decrease in the peak elastic strain energy, indicating that an appropriate fiber volume ratio was beneficial to the inhibition of the crack penetration velocity in the rubber concrete.

#### 3.2.4. Law of Energy Conversion

**Figure 10:**
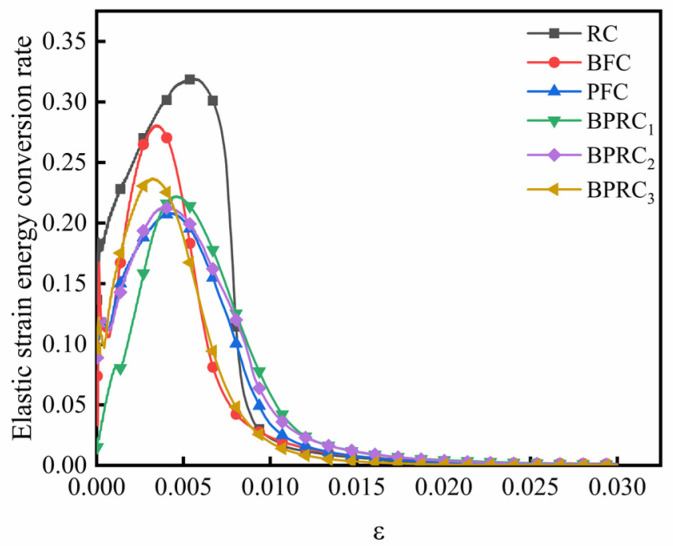
The conversion rate of elastic strain energy.

**Figure 11:**
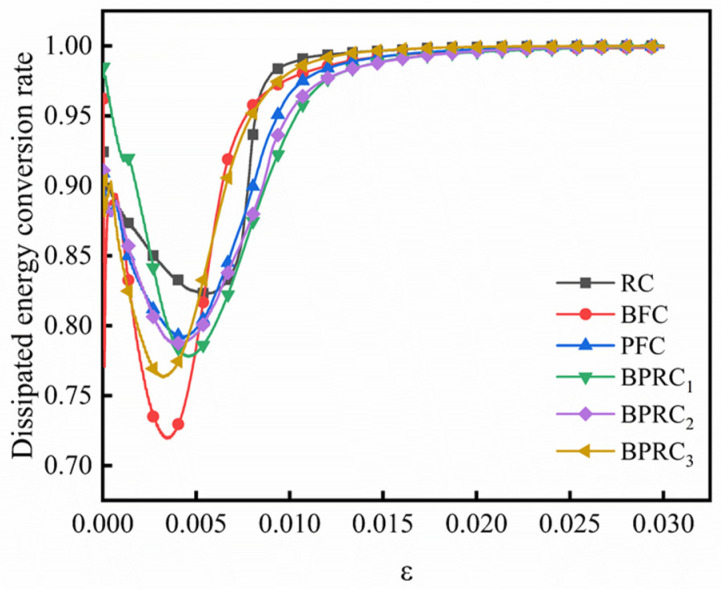
The conversion rate of dissipative energy.

The ratio of the elastic strain energy to the total strain energy represents the conversion rate of the elastic strain energy of the specimen during the process of compressive failure, and the ratio of the dissipated energy to the total strain energy represents the conversion rate of the dissipated energy of the specimen under the process of compressive failure. Figure 10 and Figure 11 respectively show the conversion rate of the elastic strain energy and the conversion rate of the dissipation energy for each group of specimens. From Figure 10 and Figure 11 together, it can be seen that, in the early stage of loading, due to the friction generated by the closure of the cracks in the rubber concrete, a certain amount of energy is consumed, so that the conversion rate of the elastic strain energy of the rubber concrete decreases and the conversion rate of the dissipative energy increases. In the elastic stage, due to the bridging effect of the fibers, the interfacial connection inside the rubber concrete is strengthened, so that the conversion rate of the elastic strain energy decreases and the conversion rate of the dissipation energy increases. After the elastic stage, the reduction rate of the elastic strain energy conversion rate and the increase rate of the dissipative energy conversion rate of the fiber rubber concrete are significantly lower than that of the RC, because the fiber can effectively inhibit the internal crack propagation speed of the rubber concrete. This shows that the fibers can effectively inhibit the brittle failure of the rubberized concrete and enhance the ductile failure characteristics.

#### 3.2.5. Stress–Strain Constitutive Model

##### Existing Constitutive Models

At present, the mechanical properties of rubber concrete/fiber–rubber concrete have been studied extensively by many scholars. However, the stress–strain constitutive model of rubber concrete/fiber–rubber concrete under uniaxial compression is seldom studied, and the failure characteristics of rubber concrete/fiber–rubber concrete are not fully understood. The four existing stress–strain constitutive models are combined (Guo model [35], Wee TH model [36], Yang KH model [37], and Carreira J model [38]), as shown in Table 3. On the basis of the stress–strain data in this paper, dimensional normalization was carried out, with MATLAB software being used to fit the stress–strain curve of each specimen.

**Figure 12:**
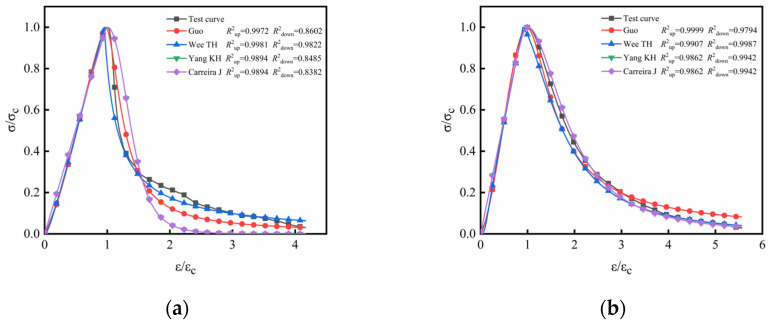

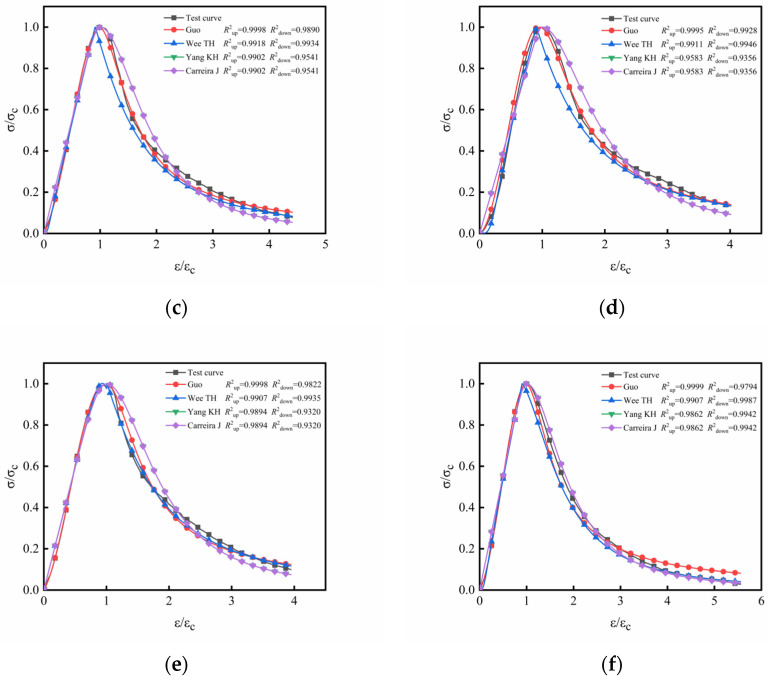
Comparison between existing constitutive models and experimental curves: (**a**) RC group; (**b**) BFC group; (**c**) PFC group; (**d**) BPRC_1_ group; (**e**) BPRC_2_ group; (**f**) BPRC3 group.

The fitting results of the stress–strain curves of each group of specimens are shown in Figure 12. It can be seen that the Gou model and Wee TH model are in good agreement with the test curve in the ascending segment, while the Yang KH model and Carreira J model overestimate the slope of the stress–strain ascending segment. In the descending section of the stress–strain curve, the agreement between the four models and the test curve varies. Among them, the Wee TH model has the highest agreement with the test curve, followed by the Guo model. The Yang KH model is close to the Carreira J model, but the Yang KH model is better than the Carreira J model on the whole. It is necessary to modify the existing constitutive model because of the low agreement between the model curve and the test curve in the stress–strain descent section.

##### Revision of the Constitutive Model

Along with the characteristics of the fiber–rubber concrete in this study, the fiber type and fiber volume rate were also introduced to establish a modified segmental fiber–rubber concrete stress–strain constitutive model. The ascending stage uses the Guo model, as shown in Equation (6), and the descending stage uses the rational fraction, as shown in Equation (7).

Ascending stage:

(6) $$y=ax+(3-2a)x^{2}+(a-2)x^{3}$$

Descending stage:

(7) $$y=\frac{bx+(c-1)x^{2}}{1+(b-2)x+cx^{2}}$$

where *x* and *y* are set according to Table 3; parameters *a*, *b*, and *c* are corrected by the peak stress, fiber type, and fiber volume ratio; and the least squares method is used to perform a regression analysis on the above parameters, with the calculation of the above parameters performed as follows in Equation (8):

(8) $$\left\{\begin{array}{l} a=0.0173\sigma_{c}-0.289\lambda -0.223\eta -0.3\\ b=-0.0021\sigma_{c}+0.341\lambda +0.263\eta +0.06\\ c=-0.0091\sigma_{c}+0.0188\lambda -0.0206\eta +1.514 \end{array}\right.$$

where *σ_c_* is the peak stress; *λ* is the volume fraction of the basalt fibers; and *η* is the volume fraction of polypropylene fibers.

**Figure 13:**
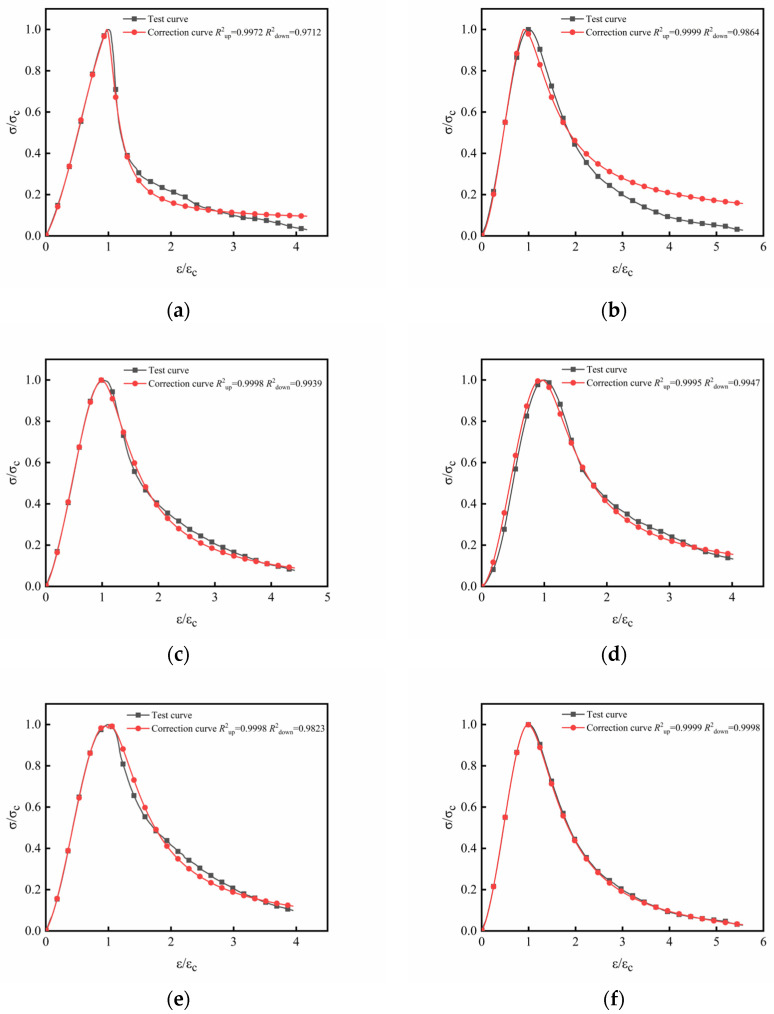
Comparison of modified constitutive model and experimental curve: (**a**) RC group; (**b**) BFC group; (**c**) PFC group; (**d**) BPRC_1_ group; (**e**) BPRC_2_ group; (**f**) BPRC_3_ group.

In order to verify the accuracy of the stress–strain correction constitutive model established in this paper, the test curve was compared with the fitting curve, as shown in Figure 13. It can be seen that the curve of the modified constitutive model established in this paper is generally consistent with the test curve, and the coincidence degree of the ascending section and descending section is greater than 0.97, which indicates that the modified constitutive model established in this paper can accurately reflect the rubber concrete and fiber–rubber concrete. Stress–strain relationship under uniaxial compression.

### 3.3. Penetration

**Figure 14:**
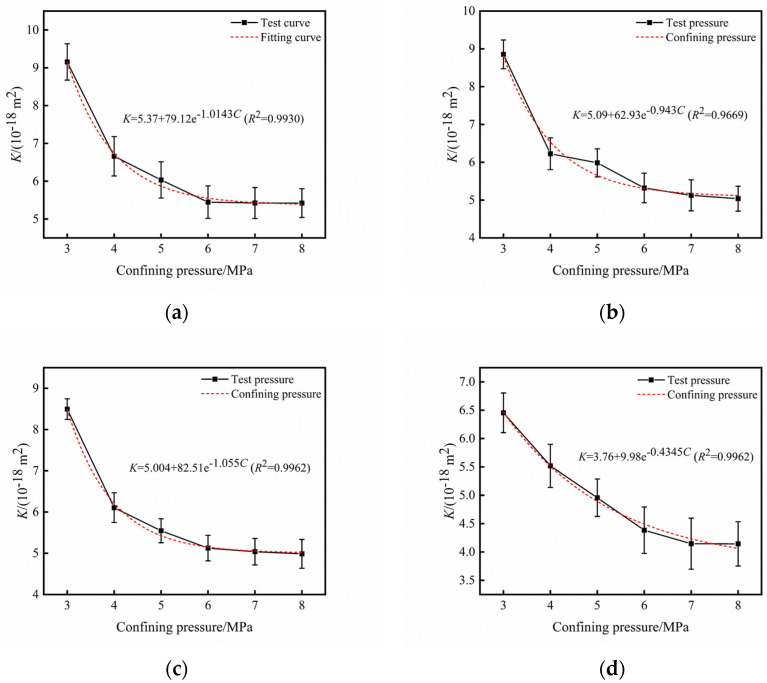

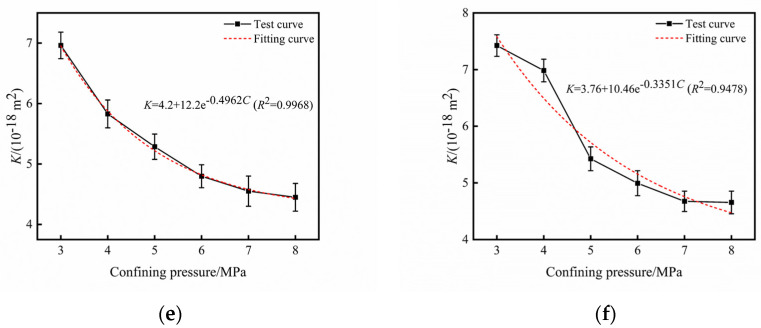
Permeability: (**a**) RC group; (**b**) BFC group; (**c**) PFC group; (**d**) BPRC_1_ group; (**e**) BPRC_2_ group; (**f**) BPRC_3_ group.

Figure 14 shows the permeability of each group of specimens under different confining pressures. It can be seen that, with the increase in the confining pressure, the permeability of each group of specimens gradually decreases, which is in line with the negative exponential decreasing relationship. When the load is 3 MPa~5 MPa, the permeability shows a sharp decreasing trend, which is mainly due to the rapid opening of the penetration channel inside the concrete with the increase in the confining pressure. When the load is 5 MPa~7 MPa, the decrease in the permeability slows down, because, with the continuous increase in the confining pressure, the pores inside the concrete become squeezed, resulting in a “short and narrow” shape. In the final stage of confining pressure loading, the permeability changes are small and tend to be stable, mainly because the pores are squeezed into the narrowest space so that the inner part of the concrete becomes compacted. Then, the permeability no longer changes with the change in the confining pressure. Under the same confining pressure, the permeability of the fiber–rubber concrete is lower than that of the RC, because the fiber can effectively make up the internal defects of the rubber concrete, enhance the compactness of the rubber concrete, and improve the microstructure, effectively inhibiting the proliferation or expansion of osmotic channels. As can be seen from Figure 14, the effect of the basalt fiber on reducing the permeability of the rubber concrete is lower than that of the polypropylene fiber, and the effect of the mixed fiber on reducing the permeability of the rubber concrete is better than that of a single fiber. In the BRPC_1_, BPRC_2_, and BPRC_3_ experiments, under the same confining pressure, the permeability of the polypropylene fiber increased gradually with the decrease in the volume fraction of the polypropylene fiber, thus also indicating that the polypropylene fiber is better than the basalt fiber at reducing the permeability of the rubberized concrete.

## 4. Conclusions

In this study, a new green and sustainable concrete (BPRC) was designed using rubber particle, basalt fiber, and polypropylene fiber and the effects of the fiber type/volume ratio on the workability, mechanical properties, and anti-permeability of the rubberized concrete were systematically studied. The main conclusions can be summarized as follows:

(1) Both fibers reduce the slump of rubberized concrete, but the basalt fiber has a greater effect on the slump of the rubberized concrete than the polypropylene fiber. At the same curing time, the water absorption of the fiber–rubber concrete is lower than that of the RC, and the effect of the polypropylene fiber on the water absorption of the rubberized concrete is greater than that of the basalt fiber.
(2) The fiber type/volume ratio has a significant effect on the RC peak stress, and the order of the effect in terms of fiber type is basalt fiber > polypropylene fiber. When the ratio of *V*_BF_ to *V*_PF_ is 1:1, the peak stress increases by 23.24% compared to that of the RC. The elastic modulus of the fiber–rubber concrete is larger than that of the RC, and the influence of the polypropylene fiber on the elastic modulus is limited. The elastic modulus of the PFC group is increased by 10.36% compared with the RC group.
(3) The total strain energy and dissipation energy of the fiber–rubber concrete are obviously larger than those of the RC. Before the peak elastic strain energy, the fiber increases the elastic strain energy growth rate of the rubberized concrete, and, after the peak elastic strain energy, the fiber reduces the elastic strain energy reduction rate of the rubberized concrete, leading to obvious ductile failure characteristics.
(4) Along with the characteristics of the fiber–rubber concrete in this paper, two parameters, namely the fiber type and fiber volume ratio, were introduced to establish a modified segmental stress–strain constitutive model. The model calculation results were in good agreement with the experimental results and can effectively reflect the effect of the fiber type/volume ratio on the constitutive behavior of the rubberized concrete.
(5) The effect of the single-mixed basalt fiber on reducing the permeability of the rubber concrete is lower than that of the single-mixed polypropylene fiber, and the effect of the mixed fiber on reducing the permeability of the rubberized concrete is better than that of the single-mixed fiber.

## Figures and Tables

**Table 1:** Main performance parameters of basalt fiber and polypropylene fiber.

Fiber	Tensile Strength/MPa	Elastic Modulus/GPa	Density/(g/cm^3^)	Diameter/µm	Length/mm	Elongation at Break/%
BF	3500	90	2.8	12	12	3.2
PF	320	0.4	0.9	8	8	38

**Table 2:** Mixing ratio (Unit: kg/m^3^).

Specimens	Cement	Sand	Stone	Water	Water Reducer	Rubber	BF	PF
RC	440	665	1040	180	4.6	0	0	0
BFC	440	594.1	1040	180	4.6	70.9	3.5	0
PFC	440	594.1	1040	180	4.6	70.9	0	1.125
BPRC_1_	440	594.1	1040	180	4.6	70.9	1.4	0.9
BPRC_2_	440	594.1	1040	180	4.6	70.9	2.1	0.675
BPRC_3_	440	594.1	1040	180	4.6	70.9	2.8	0.45

**Table 3:** Existing stress–strain constitutive models of concrete under uniaxial compression.

Models	Fitting Formula	Key Parameters
Guo model	Ascent:$y=ax+(3-2a)x^{2}+(a-2)x^{3}$ Descent: $y=\frac{x}{b{\left(x-1\right)}^{2}+x}$	*a* is the ratio of the initial elastic modulus to the secant elastic modulus; *b* according to the strength of the concrete grades and constraints determined
Wee TH model	$y=\frac{k_{1}\beta x}{k_{1}\beta -1+x^{k_{2}\beta }}$	If $f_{c}^{\prime }$ ≤ 50 MPa, $k_{1}=k_{2}=1$; If 50 MPa ≤ $f_{c}^{\prime }$ ≤ 120 MPa, $k_{1}={\left(50/f_{c}^{\prime }\right)}^{3}$, $k_{2}={\left(50/f_{c}^{\prime }\right)}^{1.3}$.
Yang KH model	$y=\frac{(\beta +1)x}{\beta +x^{\beta +1}}$	Ascent:$\beta =0.2\exp[0.73{\left(10/f_{c}\right)}^{0.67}{\left(w_{c}/2300\right)}^{1.17}]$ Descent:$\beta =0.41\exp[0.77{\left(10/f_{c}\right)}^{0.67}{\left(w_{c}/2300\right)}^{1.17}]$
Carreira J model	$y=\frac{\beta x}{\beta -1+x^{\beta }}$	$x=\varepsilon /\varepsilon_{c}$ $y=f_{c}/f_{c}^{\prime }$

## Data Availability

The data used to support the findings of this study are included within the article.
